# Prevalence and risk factors for nasal carriage of *Staphylococcus aureus* in children attending *anganwaries* (preschools) in Ujjain, India

**DOI:** 10.1186/1756-0500-6-265

**Published:** 2013-07-09

**Authors:** Sapna Dey, Senia Rosales-Klintz, Shobha Shouche, Jai Prakash Narayan Pathak, Ashish Pathak

**Affiliations:** 1 Department of Microbiology, Madhav Science College (MVM), Vikram University, 456010 Ujjain, India; 2 Department of Public Health Sciences (Global Health/IHCAR), Karolinska Institutet, Tomtebodavägen 18A, SE 171 77 Stockholm, Sweden; 3 Department of Pediatrics, R.D. Gardi Medical College, Surasa, 456010 Ujjain, India; 4 Department of Women and Children’s Health, International Maternal and Child Health Unit, Uppsala University, SE 751 85 Uppsala, Sweden

**Keywords:** *Staphylococcus aureus*, Nasal carriage, Preschool children, India

## Abstract

**Background:**

Children with nasal carriage of *S. aureus* play an important role in community spread of *S. aureus* and methicillin-resistant *S. aureus* (MRSA). Screening the nasal carriage isolates of *S. aureus* for antibiotic resistance patterns will provide guidelines for empiric therapy of community-acquired infections. The aim of the present study was to determine the prevalence of *S. aureus* and MRSA and it’s *in vitro* antibiotic susceptibility pattern among children in *anganwaries* (preschools) of Ujjain city India. This work is an extension to our previous publication in BMC Pediatrics (http://www.biomedcentral.com/1471-2431/10/100).

**Methods:**

A prospective study was done among children aged 1 to 6 years of age attending 100 *anganwaries* chosen purposely for the study to evenly cover the city. From each *anganwari* 10 children were randomly selected for nasal swabbing. Children having pyoderma were not included. Information on risk factors for nasal colonization was collected using a pre-tested questionnaire. Swabs from anterior nares were plated on 5% sheep blood agar. Antibiotic susceptibility tests were performed using Kirby-Bauer’s disc diffusion method according to performance standards of Clinical and Laboratory Standard Institute guidelines.

**Results:**

A total of 1002 children were included in the study. The prevalence of *S. aureus* nasal carriage was 35% (95% confidence interval CI 32.07 to 37.98) and that of MRSA nasal carriage was 29% (95% CI 24.28 to 33.88). The factors that were independently associated with nasal carriage of *S. aureus* were: “age-group” i.e. as the age increased beyond the age of 2 years the OR of nasal carriage decreased, “family size of more than 10 members” OR 2.59 (95% CI 1.53-4.37; *P* < 0.001), and protein energy malnutrition Grade 3 or 4 (OR 1.40, 95% CI 1.04-1.90; *P* = 0.026). The resistance pattern of *S. aureus* and MRSA showed resistance not only to single antibiotic class but co-resistance and multi-drug resistance was also common.

**Conclusions:**

The high rates of nasal carriage of *S. aureus* and MRSA and presence of resistance to commonly used antibiotics are disturbing. Antibiotic stewardship programmes that promote judicious use of antibiotic along with strategies to prevent community spread of *S. aureus* are urgently needed.

## Background

Worldwide there is increasing trend of community spread of methicillin-resistant *S. aureus* (MRSA) [1,2]. The dissemination of plasmid-borne *mec A* gene in the MRSA isolates makes them resistant to multiple antibiotics [3]. Preventing further spread of MRSA has become a major global public health problem [3,4]. Antibiotic use is the main determinant of extent of antibiotic resistance in a given geographical area [5,6]. The spread of multi-resistant strains of bacteria in the community is compounded with a paucity of new classes of antibiotics in the pipeline [7]. *S. aureus* is the most common bacterial cause for diverse range of infections, from folliculitis and furunculosis to life-threatening infections, including sepsis, deep abscesses, pneumonia, osteomyelitis, and infective endocarditis [2].

*S. aureus* colonises the skin and mucosae of human beings and several animal species [8]. Although multiple body sites can be colonised in human beings, the anterior nares of the nose is the most frequent carriage site for *S. aureus*[8]. The hand carriage and nasal carriage of *S. aureus* are strongly correlated [9] suggesting that contaminated hands most commonly cause the colonization of the nares. Nasal carriers can act as “cloud” individual during rhinitis, dispersing *S. aureus* into the environment [10]. Also, causal association between *S. aureus* nasal carriage and staphylococcal disease has been confirmed by many studies [2]. Therefore, it is important to study the prevalence of nasal carriage of *S. aureus* and factors associated with such carriage to prevent spread of *S. aureus* in the community. Screening the nasal carriage isolates of *S. aureus* for antibiotic resistance patterns will provide guidelines for empiric therapy of community-acquired infections.

In a study from same geographical area in India the main risk factors for nasal carriage of *S. aureus* were related to overcrowding i.e. children going to school and preschool [11]. Therefore, the aim of the present study was to determine the prevalence of *S. aureus* and MRSA and its *in vitro* antibiotic susceptibility pattern among children in *anganwaries* (preschools) of Ujjain city, Madhya Pradesh, India.

## Methods

This was a prospective study conducted during a 28-month period from January 2008 to April 2010.

### Study area and setting

The study was conducted among children attending *anganwaries* (preschools) of Ujjain city. The *anganwaris* are focal point for delivering the services under the Integrated Child Development Scheme (ICDS) run by the Ministry of Women and Child Development, Government of India. The services include a package of nutritional supplementation, immunisation, health check-up, and non-formal pre-school education to children of the age group 3–6 years and health and nutrition education to women in the age group 15–45 years. Each *anganwarie* caters to about 1000 community members and is served by a female health care worker. There were 302 *anganwaries* in urban Ujjain at the time of start of the study. We purposely choose 100 *anganwaries* to evenly cover the city.

### Pilot study

A one-month pilot study was conducted in December 2007 on 45 children from five *anganwaries* to estimate the nasal carriage rate of children attending *anganwaries* to facilitate sample size calculation and to study the feasibility of data collection. The nasal carriage rate for *S. aureus* in the pilot study was 11% (5/45).

### Sample size

Sample size calculation was done based on the pilot study and a previous study done in the same geographical area, which reported a nasal carriage rate between 5-8% [11]. Thus, assuming 5% as the basic percentage of nasal carriage in children up-to five in general population, 11% as nasal carriage in children attending *anganwaries*, requesting a 95% confidence interval for the proportion with width no higher than 15% and power of 90% the minimum sample size needed is 191. A conservative estimate of design effect of 4 was considered appropriate [12]; this gave a minimum sample size needed of 764 (191 × 4) children.

### Study population

The caregivers accompanying the children were verbally informed about the study one day prior to sample collection. The caregivers of the children who consented to participate were asked to accompany the child to *anganwaries* the next day. Ten children one to six years of age were randomly selected for nasal swabbing after obtaining written consent from their caregivers. Children having pyoderma (reported by caretakers) were not included.

The caregiver accompanying the child was interviewed and a questionnaire filled-in. The questionnaire contained children demographic characteristics, information on any acute illness in the past two weeks like acute watery diarrhoea (defined as passage of 3 or more loose or watery stools in the past 24 hours), upper respiratory tract infection (URTI) and fast breathing suggestive of pneumonia. Any current episode of upper respiratory tract infection (URTI) and acute watery diarrhoea was also recorded. The grading of protein energy malnutrition (PEM) was done using the Indian Academy of Pediatrics (IAP) classification [13] with the help of *anganwari* personals. The IAP classification is based on expected weight of the child for that age. Severe PEM is defined as grade III (expected weight for age between 51 to 60%) and grade IV (expected weight for age less than 50%), with or without oedema.

### Ethical considerations

The project officer, Ministry of Women and Child Development, Ujjain approved the study. The ethics committee of RD Gardi Medical College granted ethical approval for the study (approval number 42/2007).

### Sample collection

The child’s head was tilted back gently and steady from the chin. Sterile cotton swabs pre-wetted with sterile saline were rotated against the turbinate of both anterior nares of each participating child. Both swabs were inserted into a tube of Amies transport media with charcoal (HiMedia, Mumbai, India) and transported to the microbiology laboratory at Madhav Science College, Ujjain. A range of temperature between 4 to 8°C was maintained during transport.

### Microbiological laboratory method

In the microbiology laboratory swabs were plated on 5% sheep blood agar. Colonies of *S. aureus* identified by the typical colony morphology, Gram’s staining, biochemical tests for anaerobic utilization of glucose and mannitol, catalase production and tube coagulase test [14]. Antibiotic susceptibility tests were performed using Kirby-Bauer’s disc diffusion method according to performance standards of CLSI [15]. Screening for methicillin resistance was done using cefoxitin disk screen test and 6 μg/ml of oxacillin in Mueller-Hinton agar supplemented with NaCl (4% w/v; 0.68 mol/L) according to Clinical and Laboratory Standard Institute (CLSI) guidelines [15]. *S. aureus* ATCC 25923 was used as control strain. Due to limitation of resources the following restricted panel of antibiotics was selected: ampicillin, ceftriaxone, ciprofloxacin, levofloxacin, ofloxacin, gentamicin, doxycycline, tetracycline, co-trimoxazole and vancomycin. For both methicillin-sensitive *S. aureus* (MSSA) and MRSA we defined multi-drug resistant (MDR) isolates as those resistant to three different antibiotics groups [16].

### Statistical analysis

The data was entered in an Excel spreadsheet and then transferred to Stata 10.0 (Stata Corp. College Station, Texas, USA) software for statistical analysis. Prevalence of *S. aureus* and MRSA were estimated with 95% confidence intervals. The relationship between each variable and the outcome (nasal carriage of *S. aureus*) was explored using odds ratios (OR). Crude OR’s were calculated from two by two tables. A given variable was entered in the final multiple logistic model if the bivariate analysis yielded a *P* value less than 0.1. All the variables were adjusted for age and sex.

## Results

In the study 1002 children were enrolled from 100 preschools. Out of them 51% (n = 514) were boys and the remaining 49% were girls. Most children (37%, n = 374) belonged to the age group of 2 to 4 years of age, and to a family size of between 4 to 10 members (58%, n = 585) (Table 1).

**Table 1:** Factors independently associated with nasal carriage of *S. aureus*- results of multiple logistic regression* analysis

**Variable**	**n (%)**^**a**^	***S. aureus *nasal carriage**		**Crude OR**	**Std. Err.**	**95% CI**	***P *value**
		**Negative (%)**^**a**^	**Positive (%)**^**a**^				
**Age group**							
1 to 24 months	348 (35)	206 (32)	142 (41)	Ref	-	-	-
25 to 48 months	374 (37)	251 (38)	123 (35)	0.71	0.11	0.52-0.96	**0.028**
49 to 72 months	280 (28)	194 (30)	86 (24)	0.64	0.10	0.46-0.89	**0.009**
**Sex**							
Male	514 (51)	347 (53)	167 (48)	Ref	-	-	-
Female	488 (49)	304 (47)	184 (52)	1.25	0.16	0.96-1.63	0.084
**Family size**							
Up-to 4	347 (35)	238 (37)	109 (31)	Ref	-	-	-
>4 to 10	585 (58)	381 (58)	204 (58)	1.16	0.16	0.88-1.55	0.39
>10	70 (7)	32 (5)	38 (11)	2.59	0.69	1.53-4.37	**<0.001**
**History of any illness in the last 2 weeks**							
Yes	365 (36)	249 (38)	116 (33)	-	-	-	-
No	637 (64)	402 (62)	235 (67)	0.79	0.11	0.60-1.04	0.103
**Acute watery diarrhea**^**b**^							
Yes	59 (6)	40 (6)	19 (5)	-	-	-	-
No	943 (94)	611 (94)	332 (95)	0.87	0.25	0.49-1.53	0.639
**URTI**							
Yes	247 (25)	162 (25)	85 (24)				
No	755 (75)	489(75)	266 (76)	0.96	0.14	0.71-1.30	0.815
**PEM grade III/IV**							
No	734 (73)	462 (71)	272 (77)				
Yes	268 (27)	189 (29)	79 (23)	1.40	0.13	1.04-1.90	**0.026**

a. column percentage, ref-reference category, OR- odds ratio, CI-confidence interval.

b. Acute watery diarrhoea defined as passage of 3 or more loose or watery stools in the past 24 hours.

*A given variable was entered in the final multiple logistic model shown above if the bivariate analysis yielded a *P* value less than 0.1. All the variables were adjusted for age and sex.

Thirty-six percent (n = 365) of the children’s parents or caretaker of the children gave a history of any acute illness (like LM, URTI and fast breathing suggestive of pneumonia) in the past two weeks. Twenty-seven percent (n = 268) children had PEM grade III or IV (severe malnutrition).

### Nasal carriage of *S. aureus* and MRSA

Out of 1002 children included in the study a total of 351 children were culture positive for *S. aureus*. Thus, the prevalence of *S. aureus* nasal carriage was 35% (95% confidence interval CI 32.07 to 37.98). Out of the 351 *S. aureus* isolates, 102 isolates were methicillin resistant *S. aureus* (MRSA). Thus, the prevalence of MRSA nasal carriage was 29% (95% CI 24.28 to 33.88).

### Factors associated with nasal carriage of *S. aureus*

The factors that were independently associated with nasal carriage of *S. aureus* were: “age-group” i.e. as the age increased beyond the age of 2 years the OR of nasal carriage decreased, “family size of more than 10 members” OR 2.59 (95% CI 1.53-4.37; *P* < 0.001), and PEM Grade 3 or 4 (OR 1.40, 95% CI 1.04-1.90; *P* = 0.026) (Table 1).

### Antibiotic susceptibility pattern of *S. aureus* isolates

**Figure 1:**
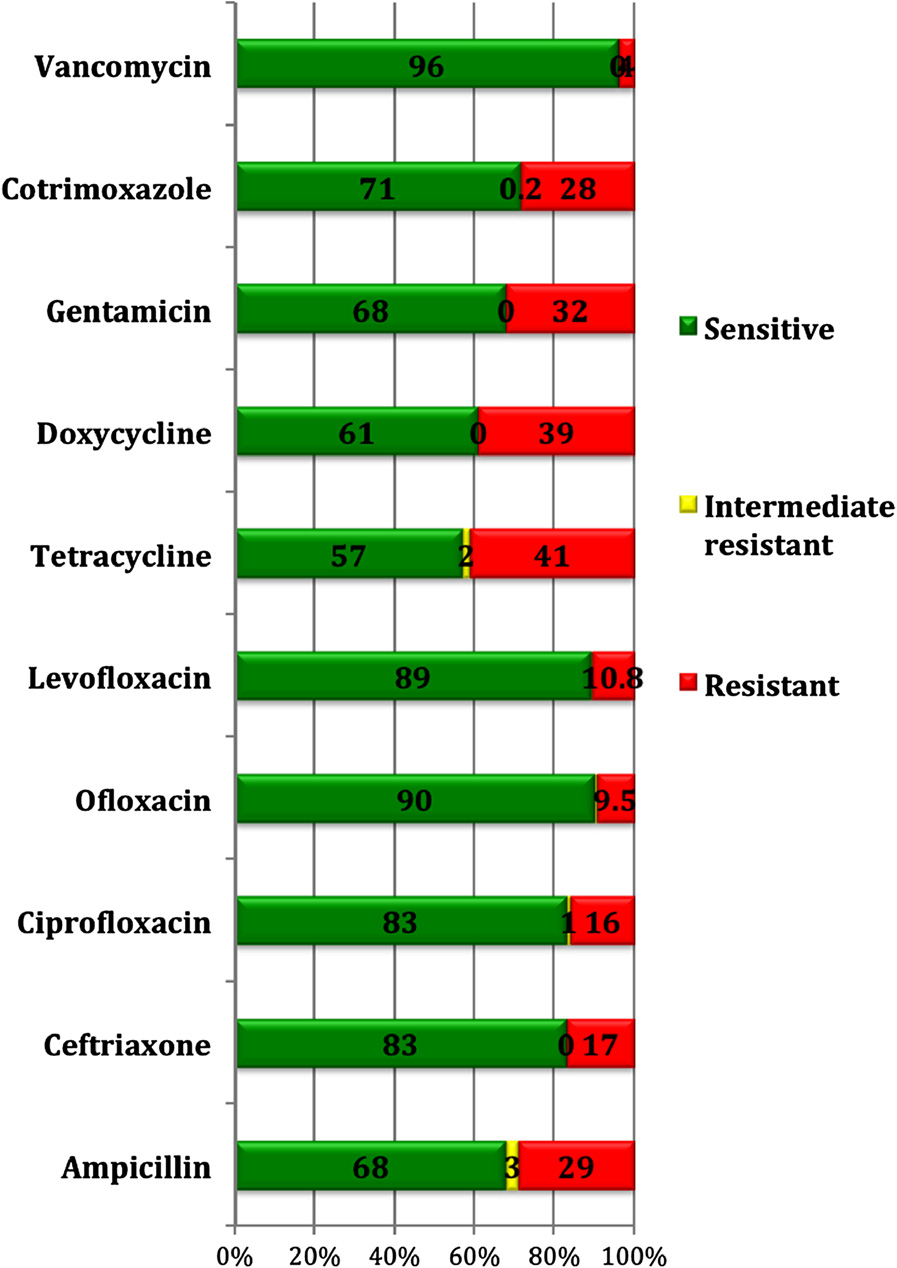
**Antibiotic susceptibility pattern of 351 *S. aureus *isolates.**

The antibiotic susceptibility pattern of *S. aureus* to individual antibiotics is shown in Figure 1. Their resistance pattern showed that co-resistance was highest for combination of tetracycline and gentamicin (30%), followed by doxycycline and gentamicin (26%) and cotrimoxazole and gentamicin (18%) (Table 2). The MDR pattern of *S. aureus* isolates is shown in Table 3. The commonest pattern of multi-drug resistance was found for a combination of gentamicin, tetracycline and cotrimoxazole (n = 62, 18%).

**Table 2:** **Co-resistance patterns (%) of 351 *S. aureus *isolates**

**Co-resistance**^**a**^	Gentamicin 114 (32)*	Ampicillin 100 (29)*	Ceftriaxone 59 (17)*	Ciprofloxacin 57 (16)*	Levofloxacin 38 (11)*
Tetracycline	**104**	54	54	52	33
144 (41)*	**(30)**	(15)	(15)	(15)	(9)
Doxycycline	**93**	50	48	47	34
137 (39)*	**(26)**	(14)	(14)	(14)	(9)
Cotrimoxazole	**63**	32	47	40	32
101 (29)*	**(18)**	(9)	(14)	(11)	(9)
Vancomycin	12	5	13	11	10
15(4)*	(3)	(1)	(4)	(3)	(3)

^a^ The co-resistance shows the pattern of resistance for the given two antibiotic groups (one in the row and other in the column). The figures in the bold represent the common pattern of co-resistance involving different antibiotics.

**Table 3:** Multi-drug resistance* pattern of 351 *S. aureus *isolates

**n**	**%**^**a**^	**Ampi**	**Cef**	**Cipro**	**Genta**	**Tetra**	**Cotri**	**Van**
62	18				**+**	**+**	**+**	
49	15			**+**	**+**	**+**		
47	14		**+**	**+**	**+**			
44	13		**+**	**+**		**+**		
39	11			**+**	**+**		**+**	
37	11		**+**	**+**			**+**	
26	7	**+**	**+**		**+**			
23	7	**+**	**+**			**+**		
22	6	**+**	**+**	**+**				
19	5	**+**	**+**				**+**	
12	3		**+**				**+**	**+**
12	3					**+**	**+**	**+**
11	3		**+**	**+**				**+**
11	3			**+**	**+**			**+**
11	3				**+**	**+**		**+**
11	3				**+**		**+**	**+**
10	3			**+**			**+**	**+**
4	1	**+**					**+**	**+**

Ampi = Ampicillin, Cef = Ceftriaxone, Cipro = Ciprofloxacin, Genta = Gentamicin, Tetra = Tetracycline, Cotri = Cotrimoxazole, Van = Vancomycin n = Number of isolates showing a given pattern %^a^- Percentage of the total (n = 351).

*Multi-drug resistance defined as resistance to at least three different antibiotic groups.

### Antibiotic susceptibility pattern of MRSA isolates

**Figure 2:**
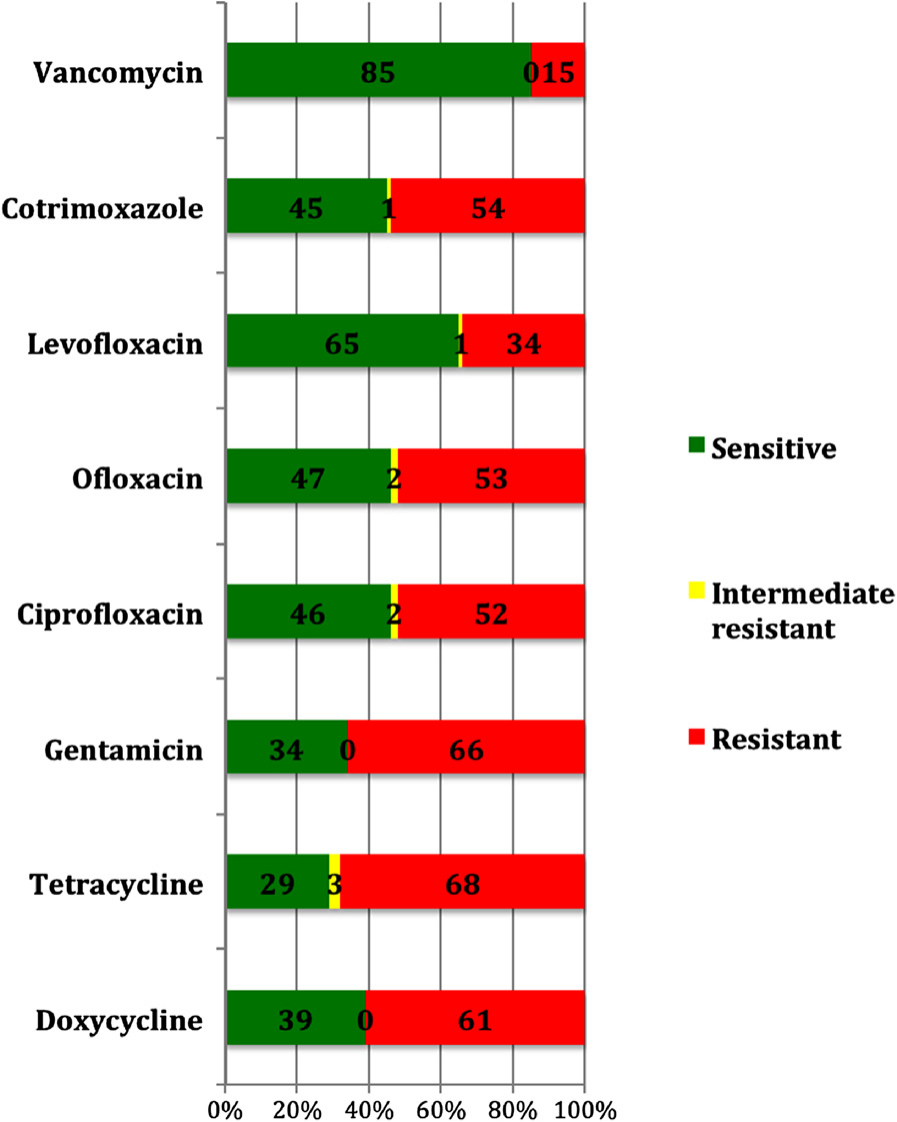
**Antibiotic susceptibility pattern of Methicillin resistant *S. aureus *(MRSA) isolates (n = 102).**

The results of antibiotic susceptibility patterns for MRSA isolates for ciprofloxacin, ofloxacin, levofloxacin, gentamicin, doxycycline, tetracycline, co-trimoxazole and vancomycin are shown in Figure 2. The commonest pattern of co-resistance was for tetracycline and gentamicin (n = 62, 61%). Table 4 shows the MDR patterns for MRSA. The commonest MDR pattern for MRSA was for a combination of doxycycline, gentamicin and cotrimoxazole (n = 44, 43%).

**Table 4:** Multi-drug resistance* pattern of 102 MRSA isolates

**Actual number of isolates**	**Doxy**	**Tetra**	**Genta**	**Cipro**	**Oflo**	**Levo**	**Cotri**	**Vanco**
44	**+**		**+**				**+**	
41	**+**		**+**	**+**				
37			**+**	**+**			**+**	
12	**+**		**+**					**+**
12	**+**						**+**	**+**
11			**+**	**+**				**+**
11			**+**				**+**	**+**
10			**+**	**+**				**+**

Doxy = Doxycline, Tetra = Tetracycline, Genta = Gentamicin, Cipro = Ciprofloxacin, Oflo = Ofloxacin, Levo = Levofloxacin, Cotri = Cotrimoxazole, Van = Vancomycin.

*Multi-drug resistance defined as resistance to at least three different antibiotic groups.

## Discussion

In this study 1002 children aged between one to six years were enrolled. The prevalence of *S. aureus* nasal carriage was 35% and that of MRSA 29%. The nasal carriage decreased as the aged increased beyond two years. Children living in larger families and those having severe PEM had higher nasal carriage of *S. aureus*. The resistance pattern of *S. aureus* and MRSA showed resistance not only to single antibiotic class but co-resistance and multi-drug resistance was also common.

A study done in the same geographical location among 1562 healthy children from 1 to 59 months of age, 6% of the children tested positive for nasal carriage of *S. aureus*[11]. The factors associated with nasal carriage were “child attending preschool” (OR 4.26, 95% CI 2.25-8.03; P = 0.007) or “school” (OR 3.02, 95% CI 1.27-7.18; P < 0.001) and “family size more than 10 members” (OR 2.76 95% CI 1.06-7.15; P = 0.03) [11]. The OR of nasal carriage in a child attending preschool were 4 times and the present study was done among the “preschool children” the prevalence is expected to be higher*.* In a community-based study done in Mangalore, southern India on 250 patients of pyoderma the nasal carriage rate of *S. aureus* and MRSA was 54% and 12% respectively. However, only half of the patients were children less than 10 years of age [17]. In another study done in north India among children of age group 5 to 15 years living in slums, the nasal carriage rate of *S. aureus* and MRSA was 52% and 4% respectively [18].

A statistically significant correlation of age with nasal carriage of *S. aureus* was noticed in our study which is similar to that reported in other low-middle income settings. In a study from Taiwan [19] the carriage rate of MRSA was higher among 2 to 6 months old children. In a study done in an elementary school in Seoul, Korea the prevalence of nasal carriers was found to be higher in younger children (≤7 years) (mean 69%) than that in older children (mean 47%) [20].

In relation to nasal carriage of *S. aureus* and sex of the child are not consistent. Another study from Taiwan showed that nasal carriage was higher for female compared to males and also increased with age [21]. In contrast a study from Lebanon showed increased risk of carriage among males [22]. Other studies have however, not found statistically significant association between sex of the child and nasal carriage of *S. aureus*[11,23].

In our study it was observed that when the family size increased the prevalence of nasal carriage of *S. aureus* increased as well. Similarly, in a study from Taiwan [19] MRSA colonization was associated with the number of children in the family (adjusted odds ratio [aOR], 1.114) and day care attendance (aOR, 1.530). Association of family size with nasal carriage of *S. aureus* is probably due to overcrowding and greater sharing of nasal flora within a large family. Prevalence of nasal carriage of MRSA has been shown to be higher among household contacts of patients with community onset MRSA disease with significant strain relatedness among the index cases and contacts [24]. Likewise, in a study done on healthy postmenopausal women in Ujjain, India one of the factor significantly associated with carriage of MDR *Escherichia coli* was a family size of more than 10 members (OR 8.23, 95% CI 2.73-24.73; p < 0.001) [25].

To our knowledge, a relationship between URTI and nasal carriage has not been studied in any Indian study before. Our study did not find any statistically significant association, however studies from other settings have reported increased spread of *S. aureus* during an episode of URTI [10].

The effect of PEM on nasal carriage of *S. aureus* in children has not been reported before. In severe PEM, acquired immunity—i.e., lymphocyte functions—as well as innate host defense mechanisms—i.e., macrophages and granulocytes—are affected [26]. Malnutrition causes atrophy of the thymus [27]. Suppression of the delayed cutaneous hypersensitivity, decreased helper T cells, impaired secretory immunoglobulin A antibody response, decreased antibody affinity, reduced concentration and activity of complement components and phagocyte dysfunction [27]. Also, there is the appearance of immature T cells in the circulation [27-29]. Because of the mechanisms discussed above malnourished children suffer in greater proportion from respiratory infections, infectious diarrhea, measles and malaria [26,28]. The infections are also characterized by a protracted course and exacerbated disease [29].

Because of the low secretory IgA levels in malnourished children the mucosal response to pathogens such as rotavirus and *E.coli* in the intestine and measles virus in the nasopharynx are found to be impaired [28]. Therefore, impaired immune response might be responsible for increased nasal carriage of *S. aureus* observed in the present study.

In the present study we found higher proportion of resistance for commonly used antibiotics then that reported in a previous study [11]. The percentage of resistance to commonly used antibiotics in our study was higher e.g. tetracycline (41%), doxycycline (39%), gentamicin (32%), ampicillin (29%) and cotrimoxazole (28%). In a study from Turkey done among 5 to 7 year-old healthy children attending day care center the resistance pattern was reported for erythromycin, clindamycin, fusidic acid, and tetracycline to be 16.6, 8.3, 5.6, and 8.3%, respectively [30]. The co-resistance pattern also demonstrates change in the resistance pattern over time in the same geographical location as the present study [11].

Antibiotic use is one of the most important determinants of antibiotic resistance [5]. A high antibiotic use rate has been reported both in the outpatients [6] and among admitted patients [31] in the same geographical area i.e. Ujjain. Antibiotic stewardship programmes that promote judicious use of antibiotic are thus urgently needed. Other strategies to prevent community spread of *S. aureus* include targeted screening among hospitalized patients based on risk factors, isolation of the carriers and decolonization.

Our study has limitations; we selected a limited pool of antibiotics for susceptibility testing and did not do molecular studies to confirm MRSA isolates due to financial constrains. We could not evaluate drugs given especially antibiotics given to the children. The dynamics of transmission of *S. aureus* in a large family needs to be studied in future using molecular diagnostics and might shed new light on the subject.

## Conclusions

The high rate of nasal carriage of *S. aureus* and MRSA and presence of resistance to commonly used antibiotics is disturbing. Antibiotic stewardship programmes that promote judicious use of antibiotic along with strategies to prevent community spread of resistant bacteria like the ReAct’s Civil Society Organization project (http://cso.reactgroup.org) are urgently needed.

## Competing interests

The authors declared that they have no competing interests.

## Authors’ contributions

SD, SS, JPNP and AP participated in the conception and design of the study. SD collected data in the field and was supervised by SS and JPNP. AP performed the statistical analysis and drafted the manuscript. JPNP and AP coordinated the study. SRK provided the external quality assurance for the data. SD, SRK, SS, JPNP and AP revised the paper critically for substantial intellectual content. All authors read and approved the final manuscript.
